# Can National Tests from the Last Year of Compulsory School Be Used to Obtain More Detailed Information about Academic Performance in Children Treated for Brain Tumours? A Nationwide, Population-Based Study from Sweden

**DOI:** 10.3390/cancers13010135

**Published:** 2021-01-04

**Authors:** Malin Lönnerblad, Eva Berglund, Ingrid van’t Hooft, Klas Blomgren

**Affiliations:** 1 Department of Special Education, Stockholm University, 106 91 Stockholm, Sweden; eva.berglund@specped.su.se; 2 Department of Women’s and Children’s Health, Karolinska Institutet, Tomtebodavägen 18 A, 171 77 Stockholm, Sweden; ingrid.hagberg-vant-hooft@sll.se; 3 Paediatric Oncology, Karolinska University Hospital, J9:30, 171 64 Stockholm, Sweden

**Keywords:** paediatric brain tumours, school performance, national tests, grades, sex, tumour grade, age at diagnosis

## Abstract

**Simple Summary:**

Children treated for brain tumours often suffer from late-appearing complications, including impaired cognitive performance. In this study, 475 Swedish children diagnosed with a brain tumour before their 15th birthday and 2197 matched controls were included. Data from compulsory national tests performed school year nine in the first foreign language English, the mother tongue Swedish and mathematics were analysed. These tests offered more detailed information on academic strengths and weaknesses than the final grades, as different skill sets were assessed. Cases performed worse than controls in English tests than in Swedish and mathematics tests, and they may have performed better in oral than written tasks. There were larger differences between girls treated for brain tumours and their female controls than between boys treated for brain tumours and their male controls. National tests may be useful to complement neuropsychological follow-ups. Characterising these shortcomings is essential to provide appropriate support and prevent social isolation.

**Abstract:**

Children treated for brain tumours often have late-appearing complications that may affect their school performance. Uneven skill profiles may help reveal late complications that can be compensated for but otherwise remain undetected. We investigated Swedish national school tests of oral, reading and writing skills in the first foreign language (English), the mother tongue (Swedish) and mathematics. Data were obtained from The Swedish Childhood Cancer Registry and Statistics Sweden. The results from 475 children diagnosed with a brain tumour before their 15th birthday and 2197 matched controls showed that children treated for brain tumours evinced more difficulties with national tests than controls in almost all subtests, especially in the subject English, and that they may perform better on oral than written tasks. There were larger differences between female cases and controls than between male cases and controls; age at diagnosis played a significant role for some subtests, whereas tumour grade did not. Missing information from national tests proved to be a strong predictor of poor academic performance. Our results show that regular educational follow-ups, as a complement to neuropsychological follow-ups, are important for all children treated for brain tumours, regardless of sex, age at diagnosis or tumour grade.

## 1. Background

The annual incidence of brain tumours in Sweden is 4.2 per 100,000 children [1]. As the survival rates nowadays have improved to more than 80% [2], a growing number of children in the school system have survived a brain tumour. Nevertheless, the tumours and treatments are very likely to lead to late-appearing complications, often called late effects, such as fatigue and cognitive deficits, the latter including reduced the attention, processing speed and working memory IQ [3,4,5]. These deficits can all affect school performance negatively [6,7,8,9]. A handful of studies have investigated the final grades in first foreign language (English); mother tongue (Finnish, Danish and Swedish, respectively) and mathematics [10,11,12,13,14]. These studies have all shown that children treated for brain tumours, hereafter termed paediatric brain tumour survivors (PBTS), are at risk of obtaining lower grades compared with controls in different school subjects. There can be many reasons for this, and thus, there is a need for more knowledge about how PBTS perform in school in order to figure out how support for the students best should be designed [15,16]. One such way is to investigate the national tests that precede the final grades. As the national tests in the first foreign language, hereafter termed English, mother tongue, hereafter termed Swedish, and mathematics assess different skills in these subjects, they potentially provide a more detailed picture of the students’ strengths and weaknesses. The main purpose of national tests in Sweden is to ascertain that the final grades will be as fair as possible across the country [17]. Thus, national tests may provide information on academic performance on both individual and group levels.

There are several reasons why we used the Swedish national tests. One reason is that, in the Swedish neuropsychological assessments, no diagnostic school-related tests are included. Another reason is that, although follow-ups in Sweden after brain tumour treatment sometimes include diagnostic school tests, they are not the same across the country. Thus, the national tests are the only tests that all children in Sweden perform. The advantage of using these tests is that they can be compared with the control group, which would not have been possible if we used diagnostic school tests performed only by the children treated for brain tumours. A third reason is the possibility to assess different skill sets (oral, reading/listening and writing) in a school setting, which we consider as adding to the ecological validity.

The main objective of this article was to investigate if results from English, Swedish and mathematics national tests could be used to identify specific academic strengths and weaknesses in PBTS beyond what can be detected from the information of final grades alone. Our questions were:

- How do PBTS perform on national tests in English, Swedish and mathematics compared with controls?
- Are there any differences in performances between the oral, reading and writing subtests in English and Swedish for the PBTS and controls, respectively?
- Are there any differences within the PBTS group due to sex, tumour grade and age at diagnosis?

We proposed that differentiated educational follow-ups should complement neuropsychological follow-ups, thereby facilitating the detection of deficits caused by the disease and the treatment, with the overall goal to provide adequate support.

## 2. Methods

### 2.1. Included Participants and Methods

**Figure 1:**
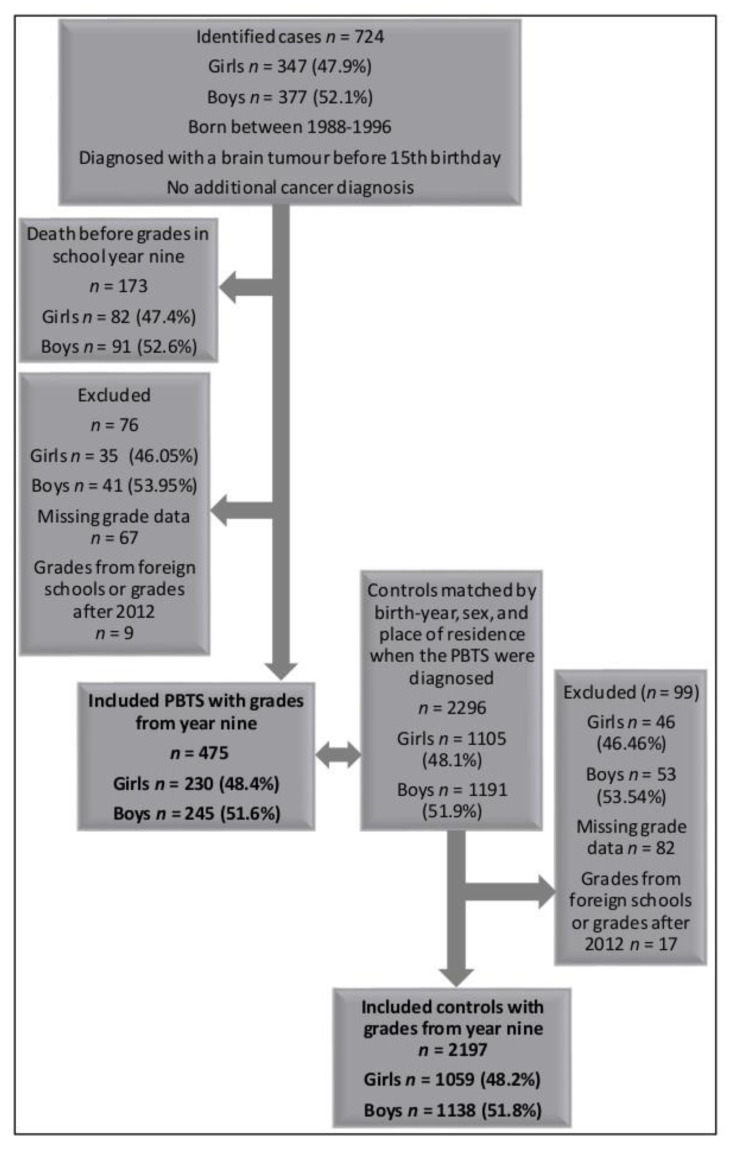
Inclusion and exclusion criteria of the paediatric brain tumour survivors (PBTS) and controls.

In this study, we explored national test results (grades from national tests) from year nine, the last year of compulsory school in Sweden, in the subjects English, Swedish and mathematics of children in Sweden born 1988–1996 and diagnosed with a brain tumour before their 15th birthday. PBTS with relapses were also included (Figure 1). 

Information about PBTS was obtained from the Swedish Childhood Cancer Registry, comprising information about 93.2% of all children in Sweden treated for cancer [18]. Coded key numbers were sent from the Swedish Childhood Cancer Registry to Statistics Sweden [19] and matched to about five controls each. From Statistics Sweden, we obtained deidentified and coded data about final grades from children included in the regular compulsory school, national test results from year nine, information about parents’ education and whether Swedish was studied as the first or second language (Table 1). PBTS were not eligible as controls, and each control only appeared for one PBTS. When we then reviewed the background factors, we found no significant differences between the PBTS group and the control group for sex (*p* = 0.931), parents’ education (mothers *p* = 0.245 and fathers *p* = 0.284) or number of children in the different groups with Swedish as the first or second language (*p* = 0.396). 

We estimated that 97% of the PBTS had completed their treatment when they obtained their grades in school year nine. Brain tumour treatment modalities include surgery, chemotherapy and radiotherapy, and any combination of these may be used, depending on the diagnosis, where all modalities typically are used in the treatment of high-grade tumours.

Within the PBTS group, we also compared females to males, children treated for high-grade tumours (WHO III-IV) to children with low-grade tumours (WHO I-II) and children diagnosed at different ages, sorted in age groups. The age groups follow the stages in the Swedish school system. Ages 0–5 are the years before school starts, ages 6–9 the first years of compulsory school and ages 10–14 are the middle and later years in compulsory school. 

In two previous articles, we investigated the final grades in the theoretical and practical subjects of this cohort [13,14]. Here, we focused on the national tests grades that precede the final grades in three theoretical subjects. 

The national tests in Sweden [20] consist of three subtests (Table 2), and these are not performed on a single occasion but over a period of several weeks.

For students in need of support, they may be allowed adaptations such as extended time or to perform the tests in multiple shorter periods [21]. We analysed the different subtests in the subjects English and Swedish. In the subject mathematics, we only had access to the composite grade of all subtests, even though this test also consists of three subtests. 

In the following, we name the three subtests of English oral, reading/listening and writing, respectively, and the three subtests of Swedish oral, reading and writing, respectively. For mathematics, we refer to the composite grades for the three subtests. The grading system in Sweden during the years of this study consisted of the grades fail, pass, pass with distinction and pass with special distinction. In all analyses, we also included what we call missing information from the national tests. Missing information is defined as PBTS or controls where we did have information about their final grades in a particular subject but, for unknown reasons, lacked information about their national test grades from one or more subtests. The national tests are compulsory for students enrolled in the regular curriculum, and it is mandatory for the schools to report the results. This means that there must be very good reasons for a child not to take one or more of the national tests or for the school not to report the results. 

### 2.2. Statistical Methods

IBM SPSS Statistics for Windows, Version 25.0. Armonk, NY, USA: IBM Corp was used for statistical analyses. We first wanted to investigate if there were overall differences between PBTS and controls for missing information and all national tests grades (fail, pass, pass with distinction and pass with special distinction). For this purpose, we used chi-square tests. For further analyses of the differences in proportions between PBTS and controls in missing information or fail, we also used chi-square test, calculating the odds ratios and 95% confidence intervals (CI). We also performed these analyses for girls and boys separately to compare female PBTS to their controls and male PBTS to their controls. To investigate if there were any differences within the PBTS group and the control group regarding performance between the three subtests in Swedish and English, respectively, we used Friedman’s ANOVA. Post hoc analyses were done with Wilcoxon signed-rank tests and a Bonferroni correction, resulting in a significance level of *p* < 0.017. These analyses could not be performed in the subject mathematics, as we only had access to the composite grade from the national tests in this subject. To detect possible differences in performances within the PBTS group due to sex or tumour grade, we ran logistic regressions with these factors as independent variables and missing information and fail as dependent variables. All models were adjusted for mothers´ and fathers’ educations, respectively, as it is well-known [22,23] that parents’ educations play an important role in children’s school performances. The interaction between sex and age at diagnosis was also tested.

## 3. Results 

### 3.1. Grades from National Tests for PBTS and Controls

There were significant differences between all national test grades of the PBTS and controls in all three subjects: English, Swedish and mathematics (Table 3).

Further analyses of the proportions between PBTS and controls showed that the odds for missing information from the national test results in the subject English were between 3.24–3.46 times higher for PBTS compared with controls (Table 4) in the subtests. For the subjects Swedish, and mathematics the odds for missing information from the national test results were between 2.49–2.94 times higher for PBTS compared with controls (Table 4). The odds for failing national tests in English were between 2.04–3.30 times higher for PBTS compared with controls for the different subtests (Table 4). For Swedish, there was a significant difference between PBTS and controls for failing only in the subtest reading, with PBTS having 1.80 times higher odds than controls, but not in the oral or writing subtests (Table 4). For mathematics, the odds to fail were 1.5 times higher for PBTS compared with controls. 

The analyses of missing information and fail for girls and boys separately revealed the same pattern as described above, with PBTS having a higher risk of missing information or failing the subtest. For most tests, there were larger differences between female PBTS and their controls than between male PBTS and their controls, especially for missing information (Table 5).

### 3.2. Missing Information from Tests and Fail as Final Grade in the Subject

When we assessed how many of the included PBTS and controls had missing information from all three subtests in English and Swedish, we found that, for the subject English, there was missing information from all three subtests for 12.8% (n = 61) of all included PBTS and 4.1% (n = 89) of all included controls. For the subject Swedish, there was missing information from all three subtests for 9.1% (n = 43) of all the included PBTS and 3.5% (n = 77) of all the included controls. For mathematics, there was missing information about the composite grades for 17.9% (n = 85) of all the included PBTS and 8.0% (n = 177) of all the included controls. We also investigated the final grade in the three different subjects for PBTS and controls with missing information from all three national tests or the composite grade in mathematics (Table 6). Missing information from all three subtests or about the composite grade seem to be a strong predictor of failing the subject. 

### 3.3. Differences between Subtests within the Groups

For the subject English, PBTS had the median grade “pass” in all three subtests, whereas controls obtained the median grade “pass with distinction” in all three subtests. However, there were statically significant differences both within the PBTS group, (χ^2^ (2) = 10.34; *p* = 0.006) and within the control group (χ^2^ (2) = 48.44; *p* < 0.001) between the different subtests. The post hoc analyses (Table 7) showed that both the PBTS and the controls performed significantly better on the oral subtest than in the subtest writing. Controls also performed significantly better on the subtest reading/listening compared with writing, but for PBTS, there was no significant differences in their performances between these two subtests. There was no significant differences in their performances between the subtests reading/listening and oral either for PBTS or for controls. 

For the subject Swedish, the PBTS obtained the median grade “pass” in all three subtests, whereas the controls obtained the median grade “pass with distinction” in the oral subtest and subtest reading and the grade “pass” in the subtest writing. There were statistically significant differences between the different subtests within the PBTS group (χ^2^(2) = 9.88; *p* = 0.007) and within the control group (χ^2^(2) = 124.06, *p* < 0.001). Post hoc analyses (Table 7) showed that both the PBTS and the controls performed significantly better on the oral subtest compared with the subtest writing. 

However, while the controls also performed significantly better on the oral subtest compared with reading and significantly better on the subtest reading compared with writing, this difference was not significant for the PBTS. For the subject mathematics, these analyses were not possible to perform, as we did not have information about the grades in the different subtests.

### 3.4. Differences within the PBTS Group Due to Sex, Tumour Grade and Age at Diagnosis

For the subject English, there were no significant differences for missing information or failing any of the subtests either for sex or for tumour grade (PBTS treated for high- or low-grade tumours). Still, the age at diagnosis was a significant factor for all subtests (Table 8). Especially, age group 0–5 but, also, 10–14 had more missing information compared with age group 6–9, but age group 6–9 failed to a larger extent compared with the other two age groups. The models were adjusted for mothers’ and fathers’ educations, respectively. Only when adjusting for the fathers’ education in the subtest reading/listening for missing information, the difference between low- and high-grade tumours became significant (OR 0.54; 0.29–0.98; *p* = 0.042). However, the odds ratio was not substantially different from the unadjusted model without the fathers’ education (Table 8). 

For the subject Swedish, there were no significant differences for missing information or failing any of the subtests between PBTS treated for high- or low-grade tumours, between girls and boys or between different age groups (Table 8). Yet, for the subtest writing, there was an interaction effect between sex and age at diagnosis for fail, with a significant difference between boys and girls in the age group 10–14 (OR 6.10; CI 1.70–21.86; *p* = 0.005) but not in the age groups 0–5 or 6–9.

For the subject mathematics, there were no significant differences for missing information or failing any of the subtests between PBTS treated for high- or low-grade tumours or between girls and boys. However, for age at diagnosis, there were significant differences; age groups 0–5 and 10–14 had more missing information compared with age group 6–9 (Table 8). For failing the national tests in mathematics, there were no significant differences for sex, tumour grade or age at diagnosis.

## 4. Discussion

The main objective of this article was to investigate if national test results in English, Swedish and mathematics could be used to identify specific academic strengths and weaknesses in children treated for brain tumours. The hypothesis was that these results could be used to identify weaknesses that cannot be detected with information only from the final grades in the subject, thereby enabling the design of suitable, individualised support. The results showed that PBTS performed significantly worse compared with controls in all subjects and evinced a higher frequency of missing information and fail, but there were considerable differences between the subtests. Previous studies [10,11,12,13,14] showed that PBTS in general have lower grades compared with controls, so this was anticipated. Compared with our study of final grades in these subjects [13], the odds ratios for PBTS to fail the national tests compared with the controls were smaller than for having fail as their final grade. For example, in mathematics, the odds ratio for fail as the final grade was 2.33 times higher for the PBTS compared with controls [13], whereas the odds ratio for fail as the composite grade at the national tests, as investigated in this study, was only 1.50 times higher for the PBTS compared with controls. The number of PBTS with missing information was two to three times higher than the number of controls with missing information, demonstrating why it was important to include and highlight missing information; it adds information that could not be deduced from the national test grades alone. 

National tests are mandatory, and there may be different explanations for the higher frequency of missing information among PBTS. Mental fatigue is a common problem for PBTS [24,25], and this may affect their capacity to participate in the national tests, which are time-consuming and may require substantial effort. Another explanation for the missing information could be that PBTS generally have high school absences [26,27,28] and, therefore, happened to be absent at the time of the tests. Missing information from the national tests seems to be a strong predictor of failing the subject, and this was true for both the PBTS and controls. As having final grades in the subjects English, Swedish and mathematics are prerequisite for qualifying to school years 10–12 in Sweden, medical follow-ups should include the question of whether the PBTS participated or not in the national tests as an indication of school performance. These school years are of particular importance, as youngsters without post-compulsory education are at higher risk of unemployment or a lower income [29] compared with those with such an education.

We expected larger differences between the PBTS and controls for the different tests and within the different subjects, as it is well-documented that children treated for brain tumours may experience neurocognitive deficits [5,30,31] that may affect their different academic skills [32,33]. One reason for the apparent lack of differences between the PBTS and controls in our study could be the grading system. During the assessed years, there were only four steps, whereas, since 2013, the Swedish grade system consists of six steps, from A to F, and it would be interesting to repeat this study with the grades from recent years and see if the differences are more easily detectable. A possible reason why differences were not found between the different subtests, or only found to a limited extent, may be that the national tests are more complex, requiring multiple neurocognitive functions simultaneously, more so than during neurocognitive testing. The national tests are also performed in school, in an environment that may be full of distractions. For example, the oral subtest in Swedish also entails listening to a text that the students are expected to discuss afterwards. This means that, in this subtest, it is not only the oral skills that are tested but, also, the auditory memory. As difficulties with memory, attention and processing speed are common [5,34], this may have had an impact on the results. Thus, neurocognitive testing at follow-ups after brain tumour treatment are important to identify specific functional neurocognitive difficulties. However, since academic follow-ups carry a component of ecological validity, we strongly recommend these be integrated in the overall assessment of PBTS short and long term. Both the PBTS and controls performed better in the verbal tests than in the writing tests in both Swedish and English. This indicates that all children in need of support could benefit from being offered the possibility of oral, rather than written, tasks and exams. 

When analysing for possible effects of sex, age at diagnosis and tumour grade, our results indicated that age at diagnosis plays a role in missing grades and failing the national tests of English and mathematics. Especially, the PBTS from the age group 0–5 and, to some extent, also age group 10–14 had more missing information compared with age group 6–9, whereas age group 6–9 failed, to a larger extent, compared with the other two age groups. It is well-known that children treated at a younger age, especially before they start school, are at risk of neurocognitive deficits [5,35]. This can be one explanation as to why this group of children had more missing information. The reason why children diagnosed at ages 10–14 had more missing information may be that these children had their diagnoses and treatments closer to the dates of the national tests and, hence, a more vivid awareness of this from both the families and teachers. These PBTS may still be suffering from the disease and the treatment in a more obvious manner than those treated years earlier. In comparison, children diagnosed at ages 6–9 were treated during the years when basic and, for most children, new skills in English and mathematics are taught in school. Our results show that they fail both the oral and the reading/listening subtests of English to a significantly larger extent compared with the other two age groups. Our interpretation is that they are considered proficient enough to participate in the tests, unlike the other two age groups who do not even take the tests, but they still fail to a large extent. 

As in our two previous studies on academic grades in theoretical [13] and practical aesthetical subjects [14], there were no significant differences between the PBTS treated for high- or low-grade tumours in their national test performance, with the exception of missing information from the subtest reading/listening in Swedish, which became significant when fathers’ educations were taken into account. While our previous studies evinced significant differences within the PBTS group between girls and boys and their final grades in most theoretical and practical aesthetic subjects [13,14], this difference was not seen for the national tests. Still, when comparing the PBTS to controls, differences between the girls and boys were detected, as the odds for missing information from the national tests or to fail the national tests were higher for female PBTS compared with their controls than for male PBTS compared with their controls. The official statistics from Sweden [36] show that girls in Sweden in general perform better than boys on the national tests. Swedish girls also have higher final grades than boys [37]. This could explain the larger differences between girls, as they perform better as a group at the baseline, and female PBTS drop to the same level as male PBTS. Despite this, we think it is important that schools find ways to also identify the girls in need of support, as extra support in general is given to boys to a larger extent than to girls in Sweden [36,38]. Therefore, there is a risk that the need for support of many girls is not discovered and may leave them at a greater disadvantage.

One way to monitor and support the academic performances of children treated for brain tumours is through school re-entry programs. These programs have been found to be beneficial for children with different disabilities [39], chronical health issues [40] or traumatic brain injuries (TBI) [41]. Moreover, programs especially designed for children treated for cancers have shown improved communication between schools, families and healthcare teams, and the programs increased teachers’ and peers’ knowledge about childhood cancer and the shortcomings that may result from the diseases and the treatments [42]. Although many school re-entry programmes have been difficult to evaluate, some have shown significant results on enhancing academic achievements and lowering the levels of depression in children with cancer [43].

In summary, our results emphasise the importance of continuous educational follow-ups. These follow-ups should be integrated into the overall assessment of PBTS during the years after the end of treatment. Moreover, information from caregivers should be communicated to schools for all children treated for brain tumours, regardless of sex, age at diagnosis or tumour grade, so that the best possible individual support can be designed. The largest differences for national test results for PBTS compared with controls were seen for the subject English, the first foreign language, so this subject should be extra closely monitored. In addition, the existence of missing information per se from any subject stands out as an important predictor of poor academic performance that may not be detected from grades alone. Finally, equal support to girls and boys is stressed. Future research should aim for more ways to evaluate different skills in multiple school subjects, including practical subjects, all aimed at providing optimal, individualised support. 

## 5. Strengths and Weaknesses

The major strength of this study is that it is a nationwide study where all children born between 1988 and 1996 and registered as treated for a brain tumour in Sweden were included. Another strength is the large number of participants and solid information about the background factors, such as parents’ educations. The main limitation is that we have no information about to what extent the children in this study had adaptations at school, such as extended time or the possibility to perform the national tests in multiple shorter periods. This information would have been valuable to detect if those children failed despite adaptations. A second limitation is that it was not possible to analyse the national tests in mathematics separately. If this was possible, it could have provided even more differentiated and valuable information. A third limitation, as discussed in Lönnerblad et al. [13,14], is the uneven proportions of excluded participants between the PBTS and controls. However, we assume that if those children treated for brain tumours from whom we had no information at all about grades were included, the differences would be even larger. It is likely that these are children with very high school absences or children attending the compulsory school for children with learning disabilities, a school system with another grading system, and information about these grades are not included in the databases of Statistics Sweden. In the general population in Sweden, this number is 1.4% [44], and one study from Sweden found that, in a group of 139 children treated for brain tumours, 3% were included in the compulsory school for children with learning disabilities [45]. Therefore, we consider that our results rather underestimate the differences between the PBTS and controls. A fourth limitation is the lack of systematically registered details about the treatments and complications of individual patients, leaving us with the tumour grade as the only useful proxy for treatment intensity, where a higher tumour grade correlates positively with a higher treatment intensity and, therefore, a higher risk of treatment-related complications.

## Figures and Tables

**Table 1:** Characteristics of the included paediatric brain tumour survivors (PBTS) (*n* = 475) and controls (*n* = 2197), including tumour classification as reported in the Swedish Childhood Cancer Registry. CNS: central nervous system.

Subject and Subtest	PBTS *N* (%)	High-Grade *N*	Low-Grade *N*	Controls *N* (%)
All	475	92	383	2197
Girls	230 (48.4)	43	187	1059 (48.2)
Boys	245 (51.6)	49	196	1138 (51.8)
Age at diagnosis (Girls)				
0–5 years	82 (35.6)	20	62	
6–9 years	51 (22.2)	13	38	
10–14 years	97 (42.2)	10	87	
Age at diagnosis (Boys)				
0–5 years	87 (35.5)	15	72	
6–9 years	66 (26.9)	10	56	
10–14 years	92 (37.6)	24	68	
Tumour classification				
Ependymomas	33 (6.9)	12	21	
Choroid plexus tumours	10 (2.1)	1	9	
Astrocytomas	172 (36.2)	10	162	
Optic nerve gliomas	41 (8.6)	-	41	
Embryonal tumours (e.g., medulloblastoma and PNET)	57 (12.0)	57	-	
Oligodendrogliomas	12 (2.5)	2	10	
Mixed and unspecified gliomas	12 (2.5)	3	9	
Neuroepithelial glial tumours of uncertain origins	4 (0.8)	-	4	
Pituitary adenomas and carcinomas	10 (2.1)	2	8	
Tumours of the cellar region (craniopharyngioma)	31 (6.5)	-	31	
Pineal parenchymal tumours	8 (1.7)	3	5	
Neuronal and mixed neuronal-glial tumours	38 (8.0)	1	37	
Meningiomas	11 (2.3)	-	11	
Specified intracranial/intraspinal tumours	1 (0.2)	-	1	
Unspecified intracranial/intraspinal tumours	8 (1.7)	-	8	
Other specified/unspecified tumours	2 (0.4)	-	2	
Nerve sheath tumours	5 (1.1)	-	5	
Germ cell tumours	8 (1.7)	1	7	
Non-CNS tumours by definition	12 (2.5)	-	12	
Swedish as the first or second language				
As the first language	450 (94.7)			2101 (95.6)
As the second language	25 (5.3)			96 (4.4)
Mothers’ education				
Low (school years 1–9 or less)	36 (7.6)			219 (9.9)
Medium (school years 10–12) ^a^	236 (49.7)			1091 (49.7)
High (higher education)	201 (42.3)			881 (40.1)
No information about education	2 (0.4)			6 (0.3)
Fathers’ education				
Low (school years 1–9 or less)	82 (17.3)			353 (16.1)
Medium (school years 10–12) ^a^	229 (48.2)			1156 (52.6)
High (higher education)	154 (32.4)			660 (30.0)
No information about education	10 (2.1)			28 (1.3)

^a^ Until 1994, school years 10–12 (equivalent to upper secondary school or high school) could be two (vocational educations) or three (theoretical educations) years in Sweden.

**Table 2:** Contents of the different subtests in the national tests of the subjects English, Swedish and mathematics.

Subject and Subtest	Description of Subtest
English—Oral Subtest A—Oral production and interaction	Discuss in English with a peer about given subjects.
English—Reading/listening Subtest B1—Reading Subtest B2 – Listening	Write answers or choose between multiple-choice answers after reading texts or listening to texts in English.
English—Writing Subtest C—Writing	Write a coherent text in English. Two topics are given to choose from.
Swedish and Swedish as a second language—Reading Subtest A—Reading and understanding	Write answers or choose between multiple-choice answers or after reading texts in Swedish.
Swedish and Swedish as a second language—Oral Subtest B—Oral	On several occasions listen to different texts in Swedish and then discuss the texts with a peer.
Swedish and Swedish as a second language—Writing Subtest C—Writing	Write a more extensive text in Swedish. Four topics are given to choose from.
Mathematics Subtest A—Oral	A group discussion with three to four students discussing a mathematical task to which each student should present a solution.
Mathematics Subtest B1—Short answer questions Subtest B2—Geometrical tasks.	Individual tasks. Calculator is not allowed.
Mathematics Subtest C— Problem solving	Individual tasks. Calculator is allowed. Complete solutions how the tasks are solved are required.

**Table 3:** Observed numbers and percentages for PBTS and controls regarding missing information (MI), fail, pass, pass with distinction (PD) and pass with special distinction (PSD) in the national tests of English, Swedish and mathematics school year 9. Chi-square comparisons between PBTS (*n* = 475) and controls (*n* = 2197) for each subtest in English and Swedish and for the composite grade in mathematics. df: degrees of freedom.

Subject Subtest			PBTS					Controls			
	MI *N* (%)	Fail *N* (%)	Pass *N* (%)	PD *N* (%)	PSD *N* (%)	MI *N* (%)	Fail *N* (%)	Pass *N* (%)	PD *N* (%)	PSD *N* (%)	Chi 2 df; *p*-Value
English											
Oral	73	44	179	127	52	115	66	830	806	380	114.98
	(15.4)	(9.3)	(37.7)	(26.7)	(10.9)	(5.2)	(3.0)	(37.8)	(36.7)	(17.3)	4; <0.001
Reading/listening	69	68	151	148	39	103	121	708	871	394	131.75
	(14.5)	(14.3)	(31.8)	(31.2)	(8.2)	(4.7)	(5.5)	(32.2)	(39.6)	(17.9)	4; <0.001
Writing	71	40	210	119	35	113	95	878	792	319	98.15
	(14.9)	(8.4)	(44.2)	(25.1)	(7.4)	(5.1)	(4.3)	(40.0)	(36.0)	(14.5)	4; <0.001
Swedish											
Oral	63	18	212	139	43	114	50	839	877	317	66.12
	(13.2)	(3.8)	(44.6)	(29.3)	(9.1)	(5.2)	(2.3)	(38.2)	(39.9)	(14.4)	4; <0.001
Reading	49	77	162	150	37	92	213	732	902	258	57.58
	(10.3)	(16.2)	(34.1)	(31.6)	(7.9)	(4.9)	(9.7)	(33.3)	(41.0)	(11.7)	4; <0.001
Writing	54	45	218	133	25	92	162	976	743	224	53.5
	(11.4)	(9.5)	(45.9)	(28.0)	(5.3)	(4.2)	(7.4)	(44.4)	(33.8)	(10.2)	4; <0.001
Mathematics											
Composite grade	85	89	184	89	28	177	292	994	524	210	60.77
	(17.9)	(18.7)	(38.7)	(18.7)	(5.9)	(8.1)	(13.3)	(45.2)	(23.9)	(9.6)	4; <0.001

**Table 4:** Odds ratio (OR), 95% confidence intervals (95% CI) and *p*-values regarding missing information and fail in the national tests and subtests in English, Swedish and mathematics year 9 for PBTS and controls. n.s. = not significant.

Subject Subtest	Missing Information OR (95% CI; *p*-Value) PBTS vs. Controls	Fail OR (95% CI; *p*-Value) PBTS vs. Controls
English		
Oral	3.29 (2.41–4.49; <0.001)	3.30 (2.22–4.89; <0.001)
Reading/listening	3.46 (2.50–4.77; <0.001)	2.87 (2.09–3.93; <0.001)
Writing	3.24 (2.36–4.44; <0.001)	2.04 (1.39–2.99, <0.001)
Swedish		
Oral	2.79 (2.02–3.87, <0.001)	1.69 (0.98–2.93; 0.075) n.s.
Reading	2.63 (1.83–3.78; <0.001)	1.80 (1.36–2.39; <0.001)
Writing	2.94 (2.06–4.17, <0.001)	1.32 (0.93–1.86, 0.130) n.s.
Mathematics		
Composite grade	2.49 (1.88–3.29: <0.001)	1.50 (1.16–1.95; 0.003)

**Table 5:** Odds ratio (OR), 95% confidence intervals (95% CI) and *p*-values for missing information and fail in the national tests and subtests in English, Swedish and mathematics year 9 for females and males, respectively. n.s. = not significant.

Subject Subtest	Missing Information OR (95% CI; *p*-Value) PBTS vs. Controls	Fail OR (95% CI; *p*-Value) PBTS vs. Controls
English		
Oral		
Girls	4.64 (3.00–7.18; <0.001)	3.63 (2.05–6.42; <0.001)
Boys	2.30 (1.46–3.64; 0.001)	3.02 (1.74–5.23; <0.001)
Reading/listening		
Girls	4.46 (2.82–7.05; <0.001)	3.22 (2.05–5.04; <0.001)
Boys	2.70 (1.70–4.27; <0.001)	2.57 (1.65–4.01; <0.001)
Writing		
Girls	4.40 (2.80–6.91; <0.001)	2.32 (1.26–4.30; 0.012)
Boys	2.44 (1.56–3.82;< 0.001)	1.88 (1.15–3.08; 0.019)
Swedish		
Oral		
Girls	3.53 (2.21–5.64; <0.001)	1.15 (0.43–3.11; 0.791) n.s.
Boys	2.23 (1.44–3.58; 0.001)	2.07 (1.06–4.03; 0.040)
Reading		
Girls	3.68 (2.23–6.07; <0.001)	2.54 (1.60–4.05; <0.001)
Boys	1.86 (1.09–3.17; 0.026)	1.52 (1.06–2.18; 0.028)
Writing		
Girls	4.16 (2.57–6.74; <0.001)	2.41 (1.32–4.39; 0.008)
Boys	2.00 (1.18–3.39; 0.016)	1.02 (0.66–1.57; 0.912) n.s.
Mathematics		
Composite grade		
Girls	3.24 (2.16–4.87; <0.001)	1.41 (0.96–2.05; 0.079) n.s.
Boys	1.98 (1.34–2.92; 0.001)	1.60 (1.11–2.30; 0.015)

**Table 6:** Information on the number and percentage of the final grades fail, pass, pass with distinction (PD) and pass with special distinction (PSD) in the school subjects English, Swedish and mathematics of the PBTS and controls with missing information on all three national test subtests or in the subjects.

Subject	PBTS	Controls
Final Grade in the Subject	*N* (%)	*N* (%)
English	61	89
Fail	45 (73.8%)	50 (56.2%)
Pass	7 (11.5%)	16 (18.0%)
PD	8 (13.1%)	12 (13.5%)
PSD	1 (1.6%)	11 (12.4%)
Swedish	43	77
Fail	22 (51.2%)	35 (45.5%)
Pass	12 (27.9%)	20 (26.0%)
PD	6 (14.0%)	17 (22.1%)
PSD	3 (7.0%)	5 (6.5%)
Mathematics	85	177
Fail	46 (54.1%)	79 (44.6%)
Pass	28 (32.9%)	73 (41.2%)
PD	6 (7.1%)	15 (8.5%)
PSD	5 (5.9%)	10 (5.6%)

**Table 7:** Post hoc analysis with Wilcoxon signed-rank tests in the subjects English and Swedish for the PBTS (*n* = 475) and controls (*n* = 2, d2197), respectively. n.s. = not significant.

Subject Subtests	PBTS *N* (%)	*p*-Value	Controls *N* (%)	*p*-Value
**English**				
oral < writing	46 (9.7%)		284 (12.9%)	
oral > writing	88 (18.5%)		432 (19.7%)	
oral = writing	341 (71.8%)		1481 (67.4%)	
		*p* = 0.004		*p* < 0.001
**English**				
reading/listening < oral	102 (21.5%)		400 (18.2%)	
reading/listening > oral	77 (16.2%)		442 (20.1%)	
reading/listening = oral	296 (62.3%)		1355 (61.7%)	
		*p* = 0.137 n.s		*p* = 0.077 n.s
**English**				
writing < reading/listening	98 (20.6%)		503 (22.9%)	
writing > reading/listening	85 (17.9%)		320 (14.6%)	
writing = reading/listening	292 (61.5%)		1374 (62.5%)	
		*p* = 0.391 n.s.		*p* < 0.001
**Swedish**				
oral < writing	70 (14.7%)		318 (14.5%)	
oral > writing	113 (23.8%)		665 (30.3%)	
oral = writing	292 (61.5%)		1214 (55.2%)	
		*p* = 0.006		*p* < 0.001
**Swedish**				
reading < oral	135 (28.4%)		598 (27.2%)	
reading > oral	102 (21.5 %)		408 (18.6%)	
reading = oral	238 (50.1%)		1191 (54.2%)	
		*p* = 0.080 n.s.		*p* < 0.001
**Swedish**				
writing < reading	103 (21.7%)		567 (25.8%)	
writing > reading	95 (20.0%)		411 (18.7%)	
writing = reading	277 (58.3%)		1219 (55.5%)	
		*p* = 0.270 n.s.		*p* < 0.001

**Table 8:** Odds ratios (OR) 95%, confidence intervals (95% CI) and *p*-values regarding missing information and fail for differences between boys and girls, high- and low-grade tumours and age groups. Interaction effect between sex and age at diagnosis is also reported. Differences between age groups are only presented when there was an overall significant difference for the age groups. n.s. = not significant.

Subject—Subtest	Missing Information OR (95% CI; *p*-Value)	Fail OR (95% CI; *p*-Value)
English–Oral		
Boys vs. girls	0.61 (0.36–1.02; 0.059) n.s.	0.88 (0.47–1.66; 0.703) n.s.
Low vs. high	0.62 (0.34–1.12; 0.115) n.s.	0.53 (0.26–1.01; 0.081) n.s.
Age groups	*p* = 0.011	*p* = 0.004
Age 0–5 vs. 10–14	1.55 (0.90–2.69; 0.117) n.s	1.32 (0.58–3.04; 0.511) n.s.
Age 6–9 vs. 10–14	0.48 (0.22–1.07; 0.073) n.s	3.34 (1.53–7.29; 0.002)
Age 6–9 vs. 0–5	0.31 (0.14–0.68; 0.003)	2.53 (1.20–5.34; 0.015)
Interaction effect	*p* = 0.453 n.s.	*p* = 0.112 n.s
English–Reading/listening		
Boys vs. girls	0.73 (0.44–1.23; 0.238) n.s.	0.83 (0.49–1.34; 0.478) n.s.
Low vs. high	0.58 (0.32–1.05; 0.070) n.s.	0.82 (0.43–1.54; 0.533) n.s.
Age groups	*p* = 0.072 n.s.	*p* = 0.007
Age 0–5 vs. 10–14	-	0.77 (0.40–1.48; 0.431) n.s
Age 6–9 vs. 10–14	-	2.085 (1.13–3.84; 0.018)
Age 6–9 vs. 0–5	-	2.72 (1.40–5.27; 0.003)
Interaction effect	*p* = 0.209 n.s.	*p* = 0.113 n.s.
English–Writing		
Boys vs. girls	0.74 (0.442–1.236; 0.248) n.s.	1.42 (0.73–2.75; 0.301) n.s.
Low vs. high	0.60 (0.33–1.09; 0.097) n.s.	0.81 (0.37–1.77; 0.600) n.s.
Age groups	*p* = 0.006	*p* = 0.279 n.s.
Age 0–5 vs. 10–14	1.92 (1.09–3.37; 0.024)	-
Age 6–9 vs. 10–14	0.64 (0.30–1.41; 0.269) n.s.	-
Age 6–9 vs. 0–5	0.34 (0.16–0.71; 0.004)	-
Interaction effect	*p* = 0.290 n.s.	*p* = 0.368 n.s.
Swedish–Oral		
Boys vs. girls	0.84 (0.49–1.44; 0.526) n.s.	2.43 (0.85–6.95; 0.099) n.s.
Low vs. high	0.59 (0.32–1.10; 0.096) n.s.	1.20 (0.34–4.28; 0.776) n.s.
Age groups	*p* = 0.236 n.s.	*p* = 0.178 n.s.
Age 0–5 vs. 10–14	-	-
Age 6–9 vs. 10–14	-	-
Age 6–9 vs. 0–5	-	-
Interaction effect	*p* = 0.975 n.s.	*p* = 0.623 n.s.
Swedish–Reading		
Boys vs. girls	0.63 (0.34–1.14; 0.128) n.s.	1.58 (0.96–2.60; 0.074) n.s.
Low vs. high	0.80 (0.39–1.65; 0.549) n.s.	0.10 (0.54–1.85; 0.995)
Age groups	*p* = 0.182 n.s.	*p* = 0.800 n.s.
Age 0–5 vs. 10–14	-	-
Age 6–9 vs. 10–14	-	-
Age 6–9 vs. 0–5	-	-
Interaction effect	*p* = 0.602 n.s.	*p* = 0.125 n.s.
Swedish–Writing		
Boys vs. girls	0.57 (0.32–1.02; 0.059) n.s.	1.62 (0.86–3.05; 0.135) n.s.
Low vs. high	0.92 (0.45–1.87; 0.807) n.s.	0.72 (0.35–1.50; 0.384) n.s
Age groups	*p* = 0.223 n.s.	*p* = 0.890 n.s
Age 0–5 vs. 10–14	-	-
Age 6–9 vs. 10–14	-	-
Age 6–9 vs. 0–5	-	-
Interaction effect	*p* = 0.845 n.s.	*p* = 0.019
Mathematics–Composite grade		
Boys vs. girls	0.87 (0.54–1.41; 0.578) n.s.	1.04 (0.65–1.66; 0.867) n.s.
Low vs. high	0.72 (0.41–1.29; 0.272) n.s.	0.68 (0.39–1.17; 0.161) n.s.
Age groups	*p* = 0.006	*p* = 0.388 n.s.
Age 0–5 vs. 10–14	1.14 (0.69–1.90; 0.601) n.s.	--
Age 6–9 vs. 10–14	0.33 (0.15–0.71; 0.005)	-
Age 6–9 vs. 0–5	0.29 (0.13–0.62; 0.002)	-
Interaction effect	*p* = 0.306 n.s.	*p* = 0.623 n.s.

## Data Availability

Data may be obtained from a third party and are not publicly available. The data that support the findings of this study are available from the Swedish Childhood Cancer Registry and Statistics Sweden. Restrictions apply to the availability of these data, which were used under license for this study.
